# Diagnostic Value of Serum Markers Combined with TVCDS in Ovarian Cancer Patients Treated with Bushen Yiqi Quyu Prescription

**DOI:** 10.1155/2021/3522906

**Published:** 2021-11-13

**Authors:** Wei Qin, Fengmin Jiang, Tao Zhang

**Affiliations:** ^1^Department of Ultrasound Medicine, Zaozhuang Mental Health Center, Zaozhuang 277103, China; ^2^Department of Ultrasound Medicine, Maternity and Child Health Care of Zaozhuang, Zaozhaung 277100, China; ^3^Department of Ultrasound Medicine, Tengzhou Central People's Hospital, Tengzhou 277599, China

## Abstract

**Background:**

To compare the diagnostic value of serum markers human epididymal protein 4 (HE4) and cyclooxygenase-1 (COX-1) combined with transvaginal color Doppler sonography (TVCDS) in ovarian cancer (OC) treated with Bushen Yiqi Quyu prescription.

**Methods:**

A total of 232 OC patients treated at the hospital from January 2018 to October 2020 were randomly divided into an observation group (*n* = 116) and control group (*n* = 116). The control group was treated with essential Western medication, and the observation group was treated with essential Western medication and Bushen Yiqi Quyu prescription. The clinical efficacy of the two groups was compared. The levels of HE4 and COX-1 were compared between the two groups before and after treatment. The ultrasonic features of TVCDS were compared between the two groups before and after treatment. The ROC curve was drawn to compare the sensitivity, specificity, and accuracy of single and combined detection of HE4, COX-1, and TVCDS in the observation group.

**Results:**

The total effective rate of the observation group was significantly higher than that of the control group. After treatment, HE4 and COX-1 levels in both groups were considerably lower than those before treatment, and in the observation group, they decreased significantly than in the control group. HE4 and COX-1 were positively correlated with the clinical stage of OC. The higher the clinical stage, the higher the levels of HE4 and COX-1. After treatment, there was no significant difference in tumor location and the boundary between the two groups. There were statistically significant differences in tumor echo, nature, morphology, calcification, internal blood flow, and lymph node metastasis, and the difference in the observation group was more evident than in control group. The sensitivity, specificity, accuracy, positive detection rate, and negative detection rate of combined detection were higher than those of single detection.

**Conclusions:**

Bushen Yiqi Quyu prescription has certain curative effects in the treatment of OC patients, which can significantly reduce the level of tumor markers and improve the symptoms of OC patients. The combined detection of HE4, COX-1, and TVCDS has high sensitivity, specificity, and accuracy, which can effectively detect OC and reduce missed diagnosis and misdiagnosis.

## 1. Introduction

Ovarian cancer (OC) is one of the common malignant tumors of the female reproductive system, and its incidence varies in the world. In China, the incidence of OC is high and on the rise, second only to endometrial cancer and cervical cancer [1]. Clinically, epithelial ovarian cancer has a high mortality rate, ranking first among female reproductive system malignant tumors [2]. The incidence of OC is insidious, and most clinically diagnosed patients are in the middle and late stages. The 5-year survival rate is only about 30%, posing a serious threat to patients' life [3]. Therefore, early screening can timely detect the disease and provide an effective treatment plan to patients. It has been reported that if patients can be diagnosed at stage I-II, the 5-year survival rate increases from 30% to more than 70% [4]. Therefore, finding an accurate and effective diagnostic method is the key to early diagnosis and treatment of OC.

HE4 is a new marker for OC. Studies have found that HE4 is highly expressed in OC cells, predominantly serous and ovarian endometrioid tumors, and currently plays an essential role in the early diagnosis, prognosis, and disease monitoring of OC [5]. COX-1 is a new ovarian tumor-associated antigen, which exists in some OC and cervical cancer cell lines. COX-1 is not expressed in healthy tissues, but is closely related to OC tissues [6]. At present, transvaginal color Doppler sonography (TVCDS) is a commonly used imaging examination method in diagnosing OC. It has the advantages of simple operation, being economical and noninvasive, and displaying the blood supply characteristics of tumors through CDFI, which is widely used in the screening and diagnosis of gynecological diseases [7].

The treatment of OC is mainly surgery, supplemented by radiotherapy and chemotherapy. However, long-term use of chemotherapy drugs can damage normal cells and cause a series of adverse reactions, seriously affecting the normal life of patients [8]. Studies in recent years have shown [9] that TCM has achieved remarkable results in adjuvant chemotherapy for tumor treatment, which can not only improve the efficacy but also alleviate the adverse reactions caused by chemotherapy and enhance the immune function of the body. In TCM, OC could be classified as “accumulation,” “abdominal mass,” and “female abdominal mass” [10]. The occurrence of OC is caused by the weakness of the body resulting in low vitality [11]. Wu et al. [12] claimed that the pathogenesis of OC was due to the deficiency of vital qi and poor blood flow, and treatment should focus on replenishing qi and removing blood stasis, with Shi Quan Da Bu decoction as the prescription. Wang Y et al. [13] reported that the etiology and pathogenesis of OC were the imbalance of Yin and Yang in the body, the obstruction of blood flow, and the accumulation of blood stasis. The treatment should be combined with the combination of strengthening and removing pathogenic factors. Bushen Yiqi Quyu prescription can not only reduce the adverse reactions of radiotherapy and chemotherapy but also enhance the immune function of the body, to improve the therapeutic effects against tumor.

The purpose of this study was to observe the diagnostic value of HE4 and COX-1 combined with TVCDS in OC patients treated with Bushen Yiqi Quyu prescription.

## 2. Materials and Methods

### 2.1. General Information

A total of 232 OC patients treated at the Tengzhou Central People's Hospital, Tengzhou, Shandong, China, from January 2018 to October 2020 were randomly divided into an observation group (*n* = 116) and control group (*n* = 116). Inclusion criteria: (1) diagnosed by histopathology; (2) met the Western medicine diagnostic criteria [14] and TCM syndrome differentiation criteria [15]; (3) have not undergone other gynecological operations; and (4) the expected survival time is more than 6 months. Exclusion criteria: (1) patients allergic to medication used in this study; (2) complicated with other gynecological diseases and benign and malignant tumors; (3) have mental or communication disorders, unable to communicate; and (4) incomplete case data. The informed consent of all patients was obtained and approved by the ethics committee of the Tengzhou Central People's Hospital, Tengzhou, Shandong, China. There was no significant difference in general data between the two groups (Table 1).

### 2.2. The Treatment

The control group was treated with prescribed Western medicine (including surgery and radiotherapy and chemotherapy in routine), and the observation group was given Bushen Yiqi Quyu prescription on the basis of the treatment in the control group. The ingredients of Bushen Yiqi Quyu prescription are 30 g Scutellariae Barbatae Herba, 30 g *Fallopia multiflora*, 30 g Dioscoreae Rhizoma, 30 Rehmanniae Radix Praeparata, 20 g *Scutellaria baicalensis*, 20 g Codonopsis Radix, 15 g Ligustri Lucidi Fructus, 15 g Chuanxiong Rhizoma, 15 g Corni Fructus, 15 g *Solanum nigrum*, 15 g Curcumae Rhizoma, 15 g Poria, 15 g Herba Hedyotidis, and 6 g Glycyrrhizae Radix et Rhizoma Praeparata cum Melle. This prescription was decocted with water and taken warm on an empty stomach in the morning and evening, 200 mL/time, once per 1 d. The treatment period was 21 d, and the treatment period was 3.

### 2.3. Observed Indicators

Clinical efficacy [16]: complete remission (CR), all lesions disappeared, and no new lesions were observed for more than 1 month; partial remission (PR), the lesions were 1/2 smaller than before chemotherapy, and there was no lesion enlargement; stable disease (SD), the lesion was without shrinkage or enlargement; and progression disease (PD), the lesions increased by 1/4 compared with before treatment, or new lesions were seen. Total effective rate was calculated using the following equation:

(1) $$\begin{array}{c} \text{Total effective rate}=\left[\frac{\left(\text{CR}+\text{PR}\right)}{\text{total number of cases}}\right]\times 100\%. \end{array}$$

### 2.4. Determination of Serum Marker Levels

5 ml fasting venous blood was collected from the two groups in the morning and made to stand at room temperature for 30 min. The serum was separated by centrifugation and stored in a refrigerator at −20°C. HE4 level was detected by enzyme-linked immunosorbent assay (ELISA) (Kananga, Sweden). The cox-1 level was measured by electrochemiluminescence (Roche). All were operated in strict accordance with the manual requirements, and all quality control met the criteria. The criteria were COX-1 > 12 U/mL was positive and HE4 > 150 pmol/L was positive.

### 2.5. TVCDS

The PHILIPS iU22 color Doppler ultrasound diagnostic instrument with a probe frequency of 7.5 MHz was used for TVCDS. The patient emptied the bladder and probed the uterus and adnexa area with an intracavitary probe through the vagina. The size and internal structure of the adnexa tumor were carefully scanned to observe the size, location, boundary, shape, and internal echo of the tumors.

### 2.6. Statistical Analysis

SPSS 22.0 software was used for statistical analysis. Measurement data were expressed by mean ± standard deviation, and comparison between groups was performed by the *t*-test. Count data were expressed by [*N* (%)], and comparison between groups was performed by the *χ*^2^ test. An ROC curve was drawn to calculate sensitivity and specificity. *p* < 0.05 was considered statistically significant.

## 3. Results

### 3.1. Comparison of Clinical Efficacy between the Two Groups

The total effective rate of the observation group was 83.62%, significantly higher than that of the control group 62.93% (Table 2). The results show that Bushen Yiqi Quyu prescription can improve the curative effects.

#### 3.1.1. Comparison of Serum Tumor Marker Levels between the Two Groups before and after Treatment

**Figure 1:**
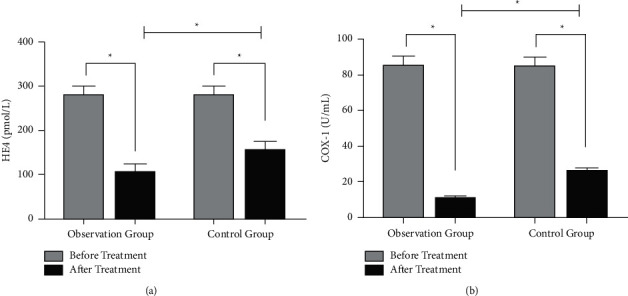
Comparison of serum tumor marker levels between the two groups before and after treatment. (a) comparison of HE4 between the two groups before and after treatment. (b) The comparison of COX-1 between the two groups before and after treatment. ^*∗*^*p* < 0.05.

Before treatment, there was no significant difference in serum HE4 and COX-1 levels between two groups. After treatment, HE4 and COX-1 of the observation group were (106.01 ± 46.58) pmol/L and (11.05 ± 3.11) U/mL, respectively. The levels of HE4 and COX-1 in the control group were (156.32 ± 47.17) pmol/mL and (26.03 ± 4.68) U/mL, respectively. After treatment, the levels of HE4 and COX-1 were significantly lower than before treatment. After treatment, the levels of HE4 and COX-1 in the observation group were lower than those in the control group (Figure 1).

#### 3.1.2. Comparison of Serum Tumor Markers in Patients with Different Stages of OC

The results showed that the levels of HE4 and COX-1 were positively correlated with the clinical stage of OC. The higher the clinical stage, the higher the levels of HE4 and COX-1 (Table 3).

#### 3.1.3. Comparison of the Ultrasonic Features in OC Patients before and after Treatment between the Two Groups

Before treatment, there was no significant difference in ultrasound indexes between the two groups. After treatment, there were statistically significant differences in echo, nature, morphology, calcification, internal blood flow, and lymph node metastasis between the two groups, and the difference in the observation group was more obvious than in the control group (Table 4).

#### 3.1.4. Comparison of the Diagnostic Value of HE4, COX-1, and TVCDS in Single and Combined Detection of OC

**Figure 2:**
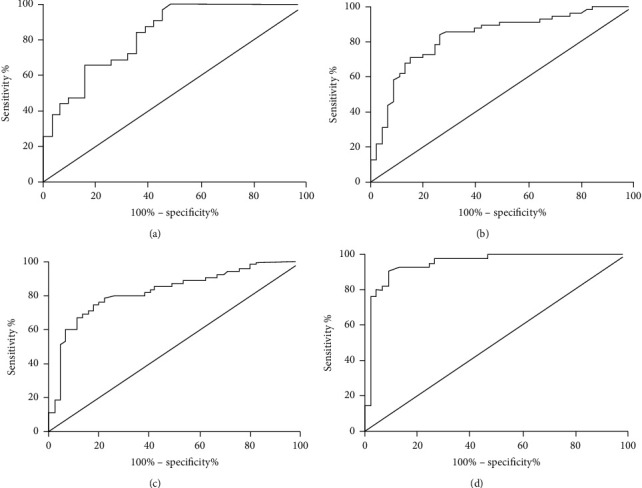
ROC curves of HE4, COX-1, and TVCDS for single and combined detection of OC. (A) ROC curve of COX-1 for the diagnosis of OC. (B) ROC curve of HE4 in the diagnosis of OC. (C) ROC curve of TVCDS in the diagnosis of OC. (D) ROC curve of the combined diagnosis of OC.

The sensitivity, specificity, accuracy, positive rate, and negative rate of COX-1 in the diagnosis of OC were 65.38%, 81.58%, 70.69%, 87.93%, and 55.17%, respectively. The sensitivity, specificity, accuracy, positive rate, and negative rate of HE4 in the diagnosis of OC were 73.08%, 84.21%, 76.72%, 90.48%, and 60.38%, respectively. The sensitivity, specificity, accuracy, positive rate, and negative rate of TVCDS in the diagnosis of OC were 67.95%, 86.84%, 74.14%, 91.38%, and 59.90%, respectively. The sensitivity, specificity, accuracy, positive rate, and negative rate of combined detection were 92.31%, 92.11%, 92.24%, 96.00%, and 85.37%, respectively. The results showed that the combined detection was higher than the single detection, and the difference in sensitivity, accuracy, and negative detection rate was statistically significant (Figure 2 and Table 5).

## 4. Discussion

OC is one of the common malignant tumors of the female reproductive system and has the highest mortality rate among gynecological tumors. As the early clinical symptoms are not apparent, it is easy to cause missed diagnosis and misdiagnosis, so most OC patients are found with the advanced stage at the time of diagnosis commonly [17]. OC is difficult to treat, with surgery and chemotherapy as the main treatment, but the prognosis is poor and the recurrence rate is high [18]. Chemotherapy has therapeutic effects, but it usually causes severe side effects. According to Chinese traditional medicine, the kidney is the innate book and the spleen is the root of acquired. The spleen and kidney can be treated together to nourish the viscera and restore vital qi [19]. If vital qi is deficient, kidney qi will become further weak and the body will produce Yin and Yang imbalance to aggravate the disease. Health professionals have different opinions about treating OC. Francisco Fernandez et al. [20] believed that invigorating the spleen and tonifying the kidney could improve the fatigue symptoms of OC patients. Tao et al. reported [21] that the treatment of OC should be based on disease differentiation, constitution differentiation, and combination of disease differentiation and syndrome differentiation.

According to our findings, the total effective rate in the observation group was significantly higher than that in the control group. HE4 and COX-1 levels in the observation group were predominantly higher than those in the control group after therapy. After treatment, there were significant differences between the two groups in tumor echo, nature, morphology, calcification, internal blood flow, and lymph node metastasis. This is because Rehmanniae Radix Praeparata, Corni Fructus, and Dioscoreae Rhizoma have a nourishing role. Corni Fructus can tonify the liver and kidney and cure frequent urination, backache, dysmenorrhea, and vaginal bleeding. Dioscoreae Rhizoma can nourish the spleen and strengthen the kidney. Rehmanniae Radix Praeparata can nourish Yin and blood. Ligustri Lucidi Fructus can nourish kidney Yin. Codonopsis Radix can tonify qi and enhance immunity. Chuanxiong Rhizoma can promote qi, activate blood, and dispel wind for dispelling pain. Solanum nigrum can remove heat and eliminate toxicity and dissipate blood stasis for detumescence. Curcumae Rhizoma can remove blood stasis and relieve pain and protect the liver and kidney. Poria can nourish the heart to tranquilize, reinforce Qi, and strengthen the spleen. Scutellariae Barbatae Herba can clear heat and remove toxicity and boost blood circulation for removing blood stasis and detumescence for relieving pain. *Fallopia multiflora* has the effect of reinforcing the kidney for supplementing essence and detoxification. Herba Hedyotidis and *Scutellaria baicalensis* can clear heat and remove toxicity and promote urination. Glycyrrhizae Radix et Rhizoma Praeparata cum Melle has the effect of benefiting qi for nourishing yin and activating Yang [22–24]. Choi et al. [25] found that *Scutellaria baicalensis* can effectively inhibit the proliferation and induce apoptosis of OC cells. Ruanet al. [26] found that *Poria cocos* extract can inhibit the occurrence and development of OC by interfering with mitochondrial function, galactose, and fatty acid metabolism. All these remedies can improve the clinical symptoms of OC patients and promote their recovery by boosting blood circulation and removing blood stasis, resolving static blood for relieving pain, diuresis, tonifying the kidney and spleen, tonifying qi, and moistening the lung.

Early screening of OC is of great significance. Serum markers play an important role in diagnostic oncology. In recent years, many potential markers have been identified and used individually or in combination to improve specificity and sensitivity, especially in the early stages of disease [27]. TVCDS is also commonly used in the diagnosis of gynecological carcinomas. It is characterized by the ability to precisely display the blood flow signals in the lesions and can directly observe the patient's ovarian morphology, size, and abnormal surrounding tissues, so as to accurately evaluate the patient's tumor. It is noninvasive, easy to operate, and economical and can be used as an important auxiliary approach for the clinical examination of OC [28, 29]. The results showed that serum HE4 and COX-1 levels increased gradually with the increase of clinical stage, indicating that the two serum markers can assist the early diagnosis of OC. In this study, the sensitivity, specificity, and accuracy of HE4 and COX-1 combined with TVCDS were significantly higher than those of either assay alone. Combined detection can further improve the diagnosis rate and reduce the rate of missed diagnosis and misdiagnosis. However, the sample size of this study is small, and the results may be biased to some extent. It is necessary to increase the sample size for further research in the future.

## 5. Conclusions

In conclusion, Bushen Yiqi Quyu prescription in this study has good therapeutic effects in treating OC, which can significantly reduce the serum indexes of patients and improve the tumor deterioration. The application of TVCDS combined with HE4 and COX-1 in the treatment of OC is optimum and significantly higher than each single detection, which has potential of clinical application for the early diagnosis of OC.

## Figures and Tables

**Table 1:** Comparison of general clinical data between the two groups.

	Observation group (*n* = 116)	Control group (*n* = 116)	*X* ^2^	*p*
Age (years)			0.075	0.784
>50	74	76		
≤50	42	40		
Weight (kg)			0.163	0.687
>62	69	72		
≤62	47	44		
The course of OC (years)			0.309	0.578
>2	37	41		
≤2	79	75		
Symptom			0.086	0.769
Obvious abdominal pain	83	85		
No obvious symptoms	33	31		
Clinical stage			0.177	0.981
I	27	29		
II	35	34		
III	43	41		
IV	11	12		
Pathological type			0.395	0.941
Serous cystadenocarcinoma	78	75		
Endometrial carcinoma	7	6		
Mucinous cystadenocarcinoma	19	22		
Others	12	13		

**Table 2:** Comparison of clinical efficacy between the two groups.

Group	*n*	CR	PR	SD	PD	Total effective rate
Observation group	116	38 (32.76)	59 (50.86)	13 (11.21)	6 (5.17)	97 (83.62)
Control group	116	21 (18.10)	52 (44.83)	27 (23.28)	16 (13.79)	73 (62.93)
*X* ^2^						14.785
*p*						0.002

**Table 3:** Comparison of serum tumor markers in patients with different stages of OC.

Clinical stage	*n*	HE4 (pmol/L)	COX-1 (U/mL)
I	56	193.52 ± 41.64	46.32 ± 8.65
II	69	227.83 ± 43.12	65.94 ± 9.28
III	84	278.96 ± 38.57	91.76 ± 11.37
IV	23	353.67 ± 32.86	136.54 ± 13.71
*t*		8.623	7.540
*p*		<0.05	<0.05

**Table 4:** Comparison of ultrasonographic features of TVCDS in OC patients before and after treatment between the two groups.

Ultrasonographic features		Observation group		Control group		*X* ^2^	*p*
		Before treatment (*n* = 116)	After treatment (*n* = 78)	Before treatment (*n* = 116)	After treatment (*n* = 95)		
Location	Above the middle	74	52	69	57	1.347	>0.05
	Below	42	26	47	38		
Echo	Hypoechoic	87	31	85	52	33.288	<0.05
	High or equal echo	29	47	31	43		
Boundary	Clarity	79	55	82	74	2.642	>0.05
	Obscure	37	23	34	21		
Characters	Solid	47	16	44	27	40.247	<0.05
	Cyst-solid	42	13	43	29		
	Cystic	27	49	29	39		
Shape	Round or oval	49	23	53	37	5.443	<0.05
	Irregular	67	55	63	58		
Calcification	No	12	25	15	23	54.532	<0.05
	Tiny	38	43	40	46		
	Bulky	66	10	61	26		
Internal blood flow	Not rich	41	53	44	51	25.605	<0.05
	Rich	75	25	72	44		
Lymph node metastasis	No	51	66	53	60	39.780	<0.05
	Yes	65	12	63	35		

**Table 5:** Diagnostic value of HE4, COX-1, and TVCDS in the single and combined detection of OC.

	Sensitivity	Specificity	Accuracy	Positive rate	Negative rate
HE4	73.08 (57/78)	84.21 (32/38)	76.72 (89/116)	90.48 (57/63)	60.38 (32/53)
COX-1	65.38 (51/78)	81.58 (31/38)	70.69 (82/116)	87.93 (51/58)	55.17 (32/58)
TVCDS	67.95 (53/78)	86.84 (33/38)	74.14 (86/116)	91.38 (53/58)	56.90 (33/58)
Combined detection	92.31 (72/78)	92.11 (35/38)	92.24 (107/116)	96.00 (72/75)	85.37 (35/41)

## Data Availability

The data used to support the findings of this study are available from the corresponding author upon request.
